# The Gut Microbiota, Kynurenine Pathway, and Immune System Interaction in the Development of Brain Cancer

**DOI:** 10.3389/fcell.2020.562812

**Published:** 2020-11-19

**Authors:** Mona Dehhaghi, Hamed Kazemi Shariat Panahi, Benjamin Heng, Gilles J. Guillemin

**Affiliations:** ^1^Neuroinflammation Group, Faculty of Medicine and Health Sciences, Macquarie University, Sydney, NSW, Australia; ^2^Pandis Community, Sydney, NSW, Australia; ^3^Department of Microbial Biotechnology, School of Biology and Centre of Excellence in Phylogeny of Living Organisms, College of Science, University of Tehran, Tehran, Iran

**Keywords:** indoleamine 2, 3-dioxygenase-1, anti-tumor T-cells, tryptophan, gut microbiota, myeloid-derived suppressor cells, kynurenine pathway, glioblastoma

## Abstract

Human gut microbiota contains a large, complex, dynamic microbial community of approximately 10^14^ microbes from more than 1,000 microbial species, i.e., equivalent to 4 × 10^6^ genes. Numerous evidence links gut microbiota with human health and diseases. Importantly, gut microbiota is involved in the development and function of the brain through a bidirectional pathway termed as the gut-brain axis. Interaction between gut microbiota and immune responses can modulate the development of neuroinflammation and cancer diseases in the brain. With respect of brain cancer, gut microbiota could modify the levels of antioxidants, amyloid protein and lipopolysaccharides, arginase 1, arginine, cytochrome C, granulocyte–macrophage colony-stimulating factor signaling (GM-CSF), IL-4, IL-6, IL-13, IL-17A, interferon gamma (IFN-γ), reactive oxygen species (ROS), reactive nitrogen species (e.g., nitric oxide and peroxynitrite), short-chain fatty acids (SCFAs), tryptophan, and tumor necrosis factor-β (TGF-β). Through these modifications, gut microbiota can modulate apoptosis, the aryl hydrocarbon receptor (AhR), autophagy, caspases activation, DNA integrity, microglia dysbiosis, mitochondria permeability, T-cell proliferation and functions, the signal transducer and activator of transcription (STAT) pathways, and tumor cell proliferation and metastasis. The outcome of such interventions could be either oncolytic or oncogenic. This review scrutinizes the oncogenic and oncolytic effects of gut microbiota by classifying the modification mechanisms into (i) amino acid deprivation (arginine and tryptophan); (ii) kynurenine pathway; (iii) microglia dysbiosis; and (iv) myeloid-derived suppressor cells (MDSCs). By delineating the complexity of the gut-microbiota-brain-cancer axis, this review aims to help the research on the development of novel therapeutic strategies that may aid the efficient eradication of brain cancers.

## Introduction

The incidence of primary brain cancer is estimated to be 7.2–12.5 per 100 million persons per year, accounting for up to 2% and 23% of all adults and childhood cancers, respectively (Wrensch et al., 2002; Marie and Shinjo, 2011). Among the primary brain tumors, astrocytoma, originates from glial cells is the most frequent brain tumor. The most malignant form of astrocytoma is glioblastoma, which has an incidence rate of 3 per 100,000 persons (Ostrom et al., 2017). Currently, the main causes of many brain tumors remain unknown. However, it has been suggested that some internal factors (i.e., genetic elements such as *POT1*) and external factors (i.e., environmental factors such as ionizing irradiation) could increase the risk of brain tumors (Picano et al., 2012; Robles-Espinoza et al., 2014).

It is well-known that some microorganisms have oncogenic or oncolytic activity on tumor cells. For example, it is estimated that up to 20% of all cancers are triggered by infectious agents (e.g., human papillomaviruses, *Helicobacter pylori*, and hepatitis B and C viruses) (De Martel et al., 2012). Interestingly, healthy individuals and cancer patients have different microbial flora in terms of population and diversity (Xuan et al., 2014). The influence of gut microbiota in various cancers has been extensively studied (Loo et al., 2017; Mehrian-Shai et al., 2019; Wong et al., 2019). However, its possible association with brain cancer is a new topic. Understanding the mechanisms of involvement of the gut microbiota-brain axis in the development or suppression of brain tumor could establish a new insight for the generation of novel anti-tumor therapeutic interventions. The gut-brain axis represents a complex multidirectional network between the gastrointestinal (GI) tract microbiota, the enteric nervous system, and the brain that influences immune responses, inflammation processes, and metabolic functions (Fung et al., 2017; Dehhaghi et al., 2019a).

The kynurenine pathway is the main route of tryptophan metabolism that results in the biosynthesis of nicotinamide adenine dinucleotide (NAD^+^) and various neuroactive intermediates (Guillemin et al., 2007; Dehhaghi et al., 2019a). In last decades, the involvement of the kynurenine pathway in brain diseases particularly brain tumors has gained more attention. Tryptophan is also catabolized through serotonin pathway that leads to biosynthesis of neuroactive metabolites such as serotonin and melatonin. It is important to note that gut microorganisms utilize tryptophan as a substrate to produce indoles indole derivatives, which are key molecules involved in signaling pathways between GI tract and immune system (Agus et al., 2018). Dysregulation of the kynurenine pathway could contribute to cancer development by disrupting the antitumoral immune response (Adams et al., 2012; Platten et al., 2019). The expression of indoleamine 2,3-dioxygenase (IDO1) and tryptophan 2,3-dioxygenase (TDO), two main enzymes contributing in tryptophan degradation, has been linked to various cancers such as melanoma, colon cancer, gynecological malignancies, lung cancer, gliomas, and bladder cancer (Théate et al., 2015; Amobi et al., 2017; Platten et al., 2019). In addition, involvement of the kynurenine pathway metabolites such as kynurenine, quinolinic acid, and 3-hydroxyanthranilic acid in cancer progression has been previously investigated (Adams et al., 2012). To the best of our knowledge, this review is the first study that comprehensively discusses the possible involvement of gut microbiota in brain cancers. The speculative mechanisms for this gut-microbiota-brain-cancer axis have been scrutinized in four sections: (i) through the kynurenine pathway; (ii) through mediation of types 1 and 2 T helper cells and the subsequent modulation of microglia (independent of the kynurenine pathway); (iii) myeloid-derived suppressor cells (MDSCs); and (iv) amino acid deprivation (i.e., arginine and tryptophan). By providing state-of-the-art information on the gut-microbiota-brain-cancer axis, this review aims to help cancer researchers and clinicians with the development of novel anti-tumor therapeutic strategies.

## The Gut Microbiota-Brain Axis

Microorganisms occur ubiquitously and have abilities to metabolize a diverse range of metabolites ranging from enzymes (Hamedi et al., 2015; Mohammadipanah et al., 2015), therapeutic lead compounds (Mohammadipanah et al., 2016; Sajedi et al., 2018), and antioxidants (Dehhaghi et al., 2018b, 2019a,c, 2020) to hydrocarbon-rich compounds, i.e., ethanol (Dehhaghi et al., 2019a; Kazemi Shariat Panahi et al., 2019b), butanol (Dehhaghi et al., 2019b), and methane (Dehhaghi et al., 2019b; Tabatabaei et al., 2019).

Human gut is one of the most dynamic niches. It contains a large and complex community of microorganisms with approximately 10^4^ microbial species, i.e., equivalent to 4 × 10^6^ genes. Interestingly, gut bacterial density has been found to be 10^12^/mL, the largest microbial density in any given ecosystem (Bhattacharjee and Lukiw, 2013; Dehhaghi et al., 2018a). Firmicutes (∼51%) and Bacteroidetes (∼48%) are the top two most abundant bacterial phyla in the human gut in respect of population, followed by other bacterial phyla including Actinobacteria, Cyanobacteria, Fusobacteria, Proteobacteria, and Verrucomicrobia (Eckburg et al., 2005; Sekirov et al., 2010; Dehhaghi et al., 2019a). These microorganisms could have a profound role in human health and diseases (Dehhaghi et al., 2018a).

Recently, it has been suggested that human gut microbiota can modulate the development and function of the central nervous system (CNS) through a bio-directional pathway termed as the gut-brain axis (Barbara et al., 2007; Foster and Neufeld, 2013; Dehhaghi et al., 2018a). The regulation of brain function occurs through modulating key processes such as neuroinflammation, neurogenesis, and neurotransmission. Microorganisms that reside in the GI tract can influence the brain activities through synthesizing neurotransmitters [i.e., dopamine, γ-amino butyric acid (GABA)] and short chain fatty acids (SCFAs), or through modulating immune responses and amino acid metabolism (Sherwin et al., 2016; Dehhaghi et al., 2019a). Many *in vivo* studies have shown that germ-free mice suffered from upregulation of genes that are associated to plasticity, steroid hormone metabolism, and synaptic long-term potentiation in the hippocampus, compared to normal mice (Buffington et al., 2016; Dehhaghi et al., 2018a; Spichak et al., 2018).

In CNS, for example, vagal afferents are responsible for transferring sensory messages (i.e., gut distension, food availability, and motor activity) to the nucleus of the solitary tract located in the brain stem (Furness et al., 2014). Neuronal inputs are subsequently sent to the higher parts of the CNS or are involved in long vago-vagal reflexes (Mulak and Bonaz, 2004). Afferent fibers in the vagus nerve direct the signals from the gut to secondary afferent neurons located in the dorsal horn. Secondary afferent neurons project to the CNS with the aid of spinothalamic pathways, which are considered to be the major pain signaling routes in the gut-brain axis (Mulak and Bonaz, 2004; Furness et al., 2014). In addition, the enteric nervous system is responsible for receiving and transmitting signals to and from the autonomic nervous system, providing a critical role in the gut-brain axis communication (Furness, 2012). A growing body of evidence has highlighted the obvious impact of the gut microbial community on the gut-brain axis functions (Quigley, 2017; Dehhaghi et al., 2018a, 2019a).

It is important to note that gut microorganisms can directly stimulate the afferent sensory neurons. Microbial SCFAs have a profound influence on enteroendocrine cells for producing various enteric neuropeptides. These can pass through the lamina propria and reach the blood stream and relevant receptors to strongly impact the extrinsic vagal innervation or enteric nervous system neurons. Bacterial circulating SCFAs, such as butyrate and propionate can bind to monocarboxylate transporters, which are extensively present at the blood-brain-barrier (BBB) and enter the CNS (Maurer et al., 2004). Monocarboxylate transporters are also expressed on the surface of neuronal and glial cells, providing a mechanism through which these compounds can be taken up and utilized as the main source of cellular energy particularly in early stages of brain development (Pellerin, 2005; Burokas et al., 2015).

It should be noted that the diversity, population, and metabolites of gut microbiota may control the state and level of both local and systemic immunity. The modulation of the immune system may be through the kynurenine pathway or through a direct effect on immune cells. For example, a bidirectional communication exists between gut microbiota and neuroendocrine system through the hypothalamic-pituitary-adrenal (HPA) axis (Farzi et al., 2018). HPA axis is a significant neuroendocrine system that controls body to ensure adequate responses to physical and psychological stressors (Smith and Vale, 2006). It has been observed that the existence of the gut microbiota in early stages of life could influence neuroendocrine responses to stress (Dinan and Cryan, 2012; O’Mahony et al., 2017). Furthermore, some disorders related to gut microbiota such as irritable bowel syndrome has a potential link with neuroendocrine system disorder such as depression. Interestingly, HPA axis is reportedly increased in both mentioned disorders (Videlock et al., 2016; Juruena et al., 2018). A reverse relationship is also true through which, for example, irritable bowel syndrome is resulted following the generation of depression due to chronic or early life stresses (Whitehead et al., 1992; Liu et al., 2017; Farzi et al., 2018). More specifically, the activation of HPA axis can increase gastrointestinal permeability and the gut microbiota composition (Heim et al., 2000; De Punder and Pruimboom, 2015). However, to this date, there is no reports on the interaction of gut microbiota in the development of brain cancer through HPA axis. In respect to brain cancer, gut-microbiota may influence the tumor microenvironment by controlling T-cell expansion and activation, microglia, cytokines production, arginine and tryptophan availability, kynurenine pathway, and ROS and antioxidants generation (Table 1).

**TABLE 1:** Some important factors in cancer that are affected by gut microbiota.

Factor	A^1^	Presence of gut microbiota			References
		**Genus/Strain**	**B^2^**	**Modulatory Mechanisms**	
GM-CSF	D^3^	*Lactobacillus reuteri, Enterococcus faecalis, Lactobacillus crispatus* and *Clostridium orbiscindens*	P^4^	Reduced expansion and activation of MDSCs by IL-17A-induced release of GM-CSF	Menetrier-Caux et al., 1998; Brown et al., 2017
IL-4	I^5^	*Lactobacillus* spp.	P	Reduced suppressive activity of MDSCs on anti-tumor T-cells by decreasing the expression of IL-4 and IL-13 and subsequent downregulation of arginase 1 expression	Rutschman et al., 2001; Bronte et al., 2003; Johansson et al., 2012
Arginine availability	−	Diverse	P	-Reduced availability of arginine through the depletion of dietary arginine in gut. -Increased radio sensitization of arginine-depleted cancer cells. -More efficient arginine deprivation therapy. -Induced autophagy and apoptosis in arginine auxotrophic cancer cells.	Syed et al., 2013; Hinrichs et al., 2018; Zou et al., 2019
Arginine and endogenous nitric oxide availability	−	*Prevotella* spp.	P	-Reduced availability of arginine and its lower subsequent conversion into nitric oxide. -Improved activity of anti-tumor T-cell by inhibiting MSDCs expansion.	Dai et al., 2015; Kao et al., 2015
Nitric oxide	−	−	P	-Conversion of dietary arginine into nitric oxide. -Increased permeability of mitochondrial membrane and subsequent promoted release of cytochrome c, expression of apoptosis inducing factor, and activation of certain caspases at high level of nitric oxide. -Nitric oxide-induced DNA damage and cell death in cancer cells. -Increased sensitization of resistant tumor cells to apoptosis during chemo-immunotherapy in the presence of nitric oxide.	Sarti et al., 2012; Bonavida and Garban, 2015; Chang et al., 2015; Tengan and Moraes, 2017
			N^6^	-Conversion of dietary arginine into nitric oxide. -Increased MSDCs expansion and subsequent decreased activity of anti-tumor T-cells.	
Peroxynitrite	I	−	N	-Conversion of dietary arginine into nitric oxide and the subsequent formation of peroxynitrite upon reaction with superoxide radicals. -Increased MSDCs-mediated suppressive activity on T-cells. -Promoted tumor progressions by rendering T-cell unresponsive due to aberrant nitration of the T-cell receptor and CD8^+^ molecules in the presence of excessive amounts of peroxynitrite.	Nagaraj et al., 2007; Gabrilovich and Nagaraj, 2009 Bentz et al., 2000; Cobbs et al., 2003; Gabrilovich and Nagaraj, 2009
Polyamines availability	−	−	N	Conversion of arginine into polyamines which may induce the proliferation and metastasis of tumor cells.	Dai et al., 2017; Zou et al., 2019
Antioxidants	−	Diverse	P	-Reduced suppressive activity of MDSCs on anti-tumor T-cells by some microbial metabolites such as vitamins, antioxidants, and polyphenols that scavenge ROS.	Dehhaghi et al., 2018a, 2019a
Tryptophan availability	−	*Burkholderia, Ralstonia, Klebsiella, Citrobacter*, and *Bifidobacterium infantis*	N	-Assimilation of dietary tryptophan which causes an aberrant proliferation and functions of effector T-cells due to the inhibition of fatty acid synthesis in human primary CD4+ T-cells by overactivating GGN2 -Induced tumor immunoresistance and survivability by the phosphorylation of eukaryotic initiation factor 2α	Ye et al., 2010; Eleftheriadis et al., 2015; Schalper et al., 2017; Kaur et al., 2019
IDO-1 IFN-γ	I	−	N	-Overactivation of AhR by tryptophan-derived metabolites of gut microbiota (e.g., kynurenine, kynurenic acid) -Ligand-activated AhR eventually increases IDO-1 by increasing the release of IL-6 and IFN-γ.	Glauben et al., 2006; DiNatale et al., 2010; Litzenburger et al., 2014; Wang et al., 2014; Dehhaghi et al., 2018a, 2019a; Martin-Gallausiaux et al., 2018; Kaur et al., 2019
			P	-Inhibited IFN-γ and IDO-1 expression by microbial SCFAs through their downregulatory effects on STAT1 and histone deacetylase	
IDO-1	I	−	N	-Increased pathogenesis of gliomas by overactivating IDO-1 through the production of inflammatory cytokines, amyloid peptide, and lipopolysaccharides. -IDO-1-suppressed expansions of T-cells and other immune cells *via* tryptophan depletion route (see above)	−
IFN-γ	I	−	N	-Weak determinant for impacting T-cell suppressive potency, accumulation, or phenotype of MDSCs − Increases cancer pathogenesis through modification of kynurenine pathway at IDO-1 step (see above).	−
Microglia dysbiosis	I	Diverse for example *Clostridium* spp.	N	-Microbial SCFAs induce the release of TGF-β that triggers dysbiosis of Th1 and Th2 in favor of microglia M2c phenotype and inhibits cytokine production, lymphocyte proliferation, and T cell differentiation.	Mantovani et al., 2004; Heijtz et al., 2011; Erny et al., 2015; Bauché and Marie, 2017; Dehhaghi et al., 2018a, 2019a,b; Roesch et al., 2018; Mehrian-Shai et al., 2019
		−	N	Induced MDSCs suppressive activity on anti-tumor T-cells due to a higher production of ROS in the brain by activated and inflamed microglia in response to microbiota-derived neurotoxic substances or metabolites (e.g., amyloid proteins and LPS)	

*^1^Anti-cancer impact by gut microbiota.*

*^2^ Effect on the brain cancer development.*

*^3^Decreased.*

*^4^Increased.*

*^5^Positive.*

*^6^Negative.*

## Amino Acid Deprivation

Gut microbiota can decrease the availability and metabolism of some dietary amino acids such as tryptophan (see Section “Tryptophan”) and arginine by utilizing them for the production of microbial protein and various metabolites (Mardinoglu et al., 2015; Dehhaghi et al., 2019a). The depletion of these amino acids could influence the progression and severity of tumor cells, including GBM.

### Arginine

Arginine is a semi-essential amino acid for humans that can be provided through diet, endogenous synthesis, and protein turnover. The human body uses arginine to produce several metabolites, some of which may affect tumors. Some pro-tumor arginine-derived metabolites in the body include polyamines and nitric oxide. Through depletion of dietary arginine, gut microbiota may decrease the metabolism of arginine-derived pro-cancer metabolites by decreasing the arginine flux in body. However, gut microbiota could also produce some arginine-derived pro-cancer metabolites during the assimilation of dietary arginine. For example, polyamines and nitric oxide are two arginine-derived metabolites that are produced by gut microbiota (Dai et al., 2015; Kao et al., 2015). The produced polyamines, then, could translocate into the brain by blood circulatory system. Following passing blood-brain-barrier, the microbial originated polyamines may induce the proliferation and metastasis of tumor cells through upregulating the expressions of ornithine decarboxylase, spermidine/spermine acetyltransferase, and Akt1 (Dai et al., 2017).

The impact of nitric oxide on cancer cells is controversial and significantly depends on its concentration, exposure time, cell type, and microenvironment. High levels of nitric oxide could suppress anti-tumor T-cell activity through enriching MDSCs (see Section “Myeloid-Derived Suppressor Cells”). Moreover, aberrant production of nitric oxide by gut microbiota could increase the flux of peroxynitrite, following its reaction with superoxide radicals. Peroxynitrite is one of the key compounds that promotes MDSC suppressive activity on T-cell function (see Section “Myeloid-Derived Suppressor Cells”). Alternatively, nitric oxide interferes with T-cell function through the inhibition of MHC class II transcription (Rivoltini et al., 2002), the inhibition of the JAK3-STAT5 signaling pathway, and the induction of T-cell apoptosis (Bingisser et al., 1998; Harari and Liao, 2004). Furthermore, high levels of nitric oxide could increase the permeability of the mitochondrial membrane that subsequently triggers the release of cytochrome c, the expression of an apoptosis inducing factor, and the activation of certain caspases (Sarti et al., 2012; Tengan and Moraes, 2017). Interestingly, cancer cells are more susceptible to cytotoxic effects (DNA and mitochondria damage) of nitric oxide due to aberrant P53 protein, compared to normal cells (Chang et al., 2015). Moreover, the sensitization of resistant tumor cells to apoptosis during chemo-immunotherapy, increases in the presence of nitric oxide (Bonavida and Garban, 2015).

It should be noted that arginine starvation can have both positive and negative effects on tumors. Like arginase 1 in MDSCs, the assimilation of L-arginine by gut microbiota will lead to its depletion from the tumor microenvironment. This phenomenon has an inhibitory effect on T-cell proliferation by suppressing T-cell cell cycle regulators (cyclin-dependent kinase 4 and cyclin D3) and downregulating the expression of T-cell antigen, i.e., CD3 zeta(ζ)-chain (Rodriguez et al., 2002, 2007). Accordingly, the failure of T-cells to upregulate the mentioned cell regulators, arrests T-cell in the G_0_-G_1_ phase of the cell cycle. This is done through aberrant downstream signaling, i.e., a blocked GCN2 signaling pathway that involves a lower level of Rb protein phosphorylation, and weak E2F1 expression and binding.

On the positive side, the depletion of nutritional arginine by gut microbiota can be beneficial for eradication of arginine auxotrophic tumors, which lack the enzyme (i.e., argininosuccinate synthetase) required for the conversion of citrulline to arginine (Feun et al., 2008; Syed et al., 2013; Khoury et al., 2015; Zou et al., 2019). Therefore, these types of tumor cells must rely on the exterior source of arginine to meet their extremely high metabolic rate and intensive growth. Even the sensitization of nonauxotrophic glioblastoma is induced in the absence of arginine (Hinrichs et al., 2018). Under arginine depletion stress, tumor cells switch to autophagy to maintain their functions, but eventually undergo apoptosis due to excessive autophagy.

### Tryptophan

Tryptophan is an essential amino acid, which means it cannot be synthesized endogenously in the human body and must be supplied through dietary intake. Two forms of tryptophan, i.e., either bound to albumin or free form, can be found in the human body (Dehhaghi et al., 2019a). Only the free form of tryptophan can across the BBB through non-specific L-amino acid transporters. Although the level of tryptophan in human tissues is lower than other amino acids, it is a critical component of several metabolic pathways. After tryptophan uptake, it participates in protein synthesis or enters various metabolic pathways based on the tissue and the enzymes expressed. While approximately, 90% of tryptophan is metabolized through the kynurenine pathway, generating various neuroactive metabolites in the body, 3–10% of tryptophan is utilized for the synthesis of chemical messengers such as serotonin, tryptamin, and other indole-derived metabolites (Palego et al., 2016; Dehhaghi et al., 2019a). Biotransformation of tryptophan and its availability is regulated by both endogenous and exogenous factors. The association of tryptophan depletion with some neurological disorders such as depression and mood-affective diseases are well-known (Kanchanatawan et al., 2018).

With respect to cancers, tryptophan depletion is increasingly being identified as a critical factor in tumor cell development. Importantly, tryptophan is highly catabolized in a microenvironment of tumor cells and inflammation regions. This local tryptophan depletion promotes T-cells to activate general control nonderepressible-2 (GCN2) kinase, i.e., a serine-threonine kinase that responds to amino acid deprivation. Consequently, a stress response is induced by activated GCN2 that leads to an aberrant proliferation of effector T-cells (Schalper et al., 2017). More specifically, activated GCN2 is able to inhibit fatty acid synthesis in human primary CD4^+^ T-cells that is essential for their regular proliferations and functions (Eleftheriadis et al., 2015). It is worth mentioning that CD4^+^ T-cells show a reduced survivability in the presence of kynurenine under tryptophan-depleted locations. In tumor cells, abnormal angiogenesis is associated with the lack of blood supply, which is directly related to the development of hypoxia and the deprivation of glucose, amino acids, and other essential nutrients. Under this condition, the phosphorylation of eukaryotic initiation factor 2α (eIF2 α) occurs by activation of GCN2, which upregulates activating transcription factor 4 (ATF4) expression, increases amino acid biosynthesis, and eventually induces tumor immunoresistance and its survival (Ye et al., 2010). Additionally, IDO-1 expressing tumor cells respond to tryptophan shortage by expressing amino acid transporter genes (i.e., *SLC7A11, SLC1A4*, and *SLC1A5*). The upregulation of ATF4-dependent expression of *SLC1A5* and its splice variants eventually increase glutamine and tryptophan uptake that are highly demanded for rapid amino acids synthesis in tumor cells (Timosenko et al., 2016).

Expression of IDO-1 in non-antigen-presenting cells, such as tumor cells, promotes the escape of the tumors from immunosurveillance (Munn and Mellor, 2007). Generally, IDO-1 can inhibit glucose uptake, glycolysis, and glutaminolysis, which contribute in its immunosuppressive activity (Eleftheriadis et al., 2013). Like activated T cells, highly proliferating cells such as cancer cells change their metabolic pathways from pyruvate oxidation to the glycolytic and glutaminolytic pathways. Cancerous cells showed Warburg’s phenomenon in which is an enhanced cytoplasmic glycolysis/mitochondrial oxidation ratio (Warburg, 1956; Wang et al., 2011). Most cancer cells can directly or indirectly affect the function of p53 (a potent tumor suppressor) that inhibits cell proliferation by arresting the cell cycle in G1-phase. In addition, p53 plays significant role in the modulation of cellular metabolism through inhibiting glucose uptake and aerobic glycolysis (Brady and Attardi, 2010; Shen et al., 2012). With respect to the similarity in glucose metabolism in activated T cells and cancer cells, it has been shown that IDO-induced tryptophan depletion increased p53 level. Both IDO-1 and p53 inhibited the aerobic glycolysis in alloreactive T cells (Eleftheriadis et al., 2014). IDO-induced tryptophan depletion also activated the GCN2 kinase leading to p53 up-regulation in T cells (Eleftheriadis et al., 2014).

**FIGURE 1:**
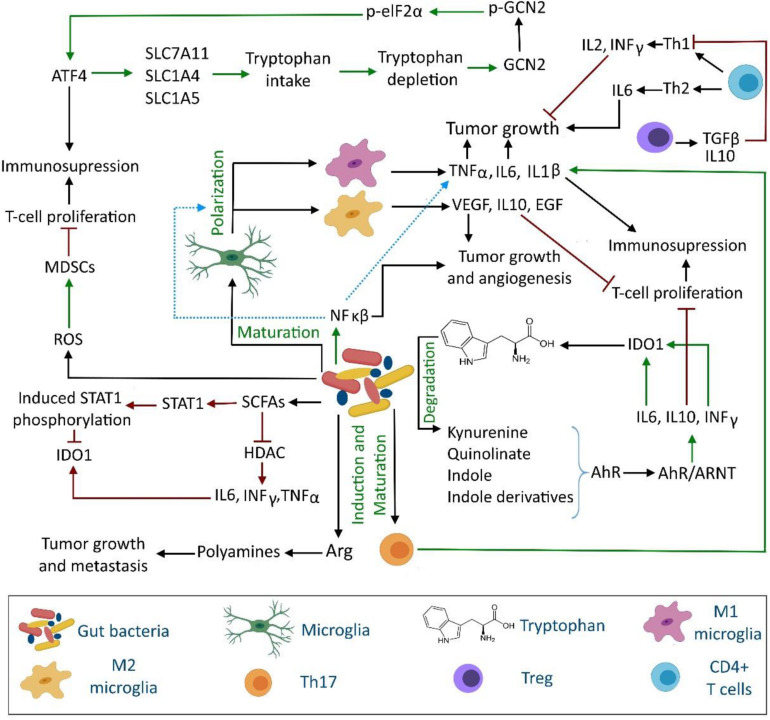
Interaction between gut microbiota, immune system and tryptophan metabolism in brain tumor development. The gut microbiota can influence the brain tumor cells proliferation and metastasis through direct and indirect regulation of tryptophan and arginine metabolism, inflammatory cytokine production, release of short chain fatty acids, aryl hydrocarbon receptor modulation, microglia maturation, and T cell proliferation. AhR, aryl hydrocarbon receptor; Arg, arginine; ARNT, AhR nuclear translocator; ATF4, activating transcription factor 4; eIF2α, eukaryotic initiation factor 2α (eIF2 α); GCN2, general control nonderepressible-2; HDAC, histone deacetylase; MDSCs, myeloid-derived suppressor cells; ROS, reactive oxygen species; SCFAs, short chain fatty acids; STAT, signal transducer and activator of transcription.

Based on *in silico* analyses, bacterial phyla residing in the human GI tract including Actinobacteria, Bacteroides, Firmicutes, Fusobacteria, and Proteobacteria possess complex pathways to metabolize tryptophan and produce neuroactive metabolites such as kynurenine, kynurenic acid, quinolinate, tryptamine, indole, and indole-derivatives (DiNatale et al., 2010; Kaur et al., 2019). Some gut bacterial genera (e.g., *Burkholderia*, *Ralstonia*, *Klebsiella*, and *Citrobacter*) have higher potential to convert tryptophan to neuroactive compounds, compared to other bacteria (Kaur et al., 2019). Interestingly, microbial tryptophan-derived indole and indole derivatives could profoundly modulate gut homeostasis and cellular gene expression. Moreover, indolyl metabolites may be significant signaling molecules between the gut and immune system as they can bind to the aryl hydrocarbon receptor (AhR) and activate it locally and systemically (Cheong and Sun, 2018; Dehhaghi et al., 2019a). More importantly, gut-microbiota can affect the disease state of cancer by modulating IDO-1 activity (see Section “Kynurenine Pathway”) through regulating AhR activity. The interaction of some tryptophan-derived gut microbiota with AhR causes its activation. Ligand-activated AhR then regulates the functions of a wide range of innate and adaptive immune system such as dendritic cells, natural killer cells, macrophages, regulatory T-cells, and type 17 and 22 helper T-cells (Cheong and Sun, 2018). The dissociation of AhR from the chaperone heat shock protein 90 occurs after binding ligands to AhR. The ligand-activated AhR translocates nucleus and forms a heterodimeric complex with the AhR nuclear translocator (ARNT) protein. The AhR-ARNT complex is a transcription factor that regulates the expression of IL-6 in macrophages, IL-10 in natural killer cells and dendritic cells (Litzenburger et al., 2014; Wang et al., 2014). IL-6 then activates IDO-1 and indirectly contributes in increased kynurenine and kynurenic acid production and AhR activation. Moreover, AhR activation in natural killer cells induces INF-γ production that subsequently induces IDO-1 expression and eventually leads to tryptophan depletion (Figure 1).

Overall, the depletion of tryptophan has pro-cancer activities (Table 1) and increases the survivability and severity of tumors. In a tryptophan-depleted microenvironment, the responses of antigen-specific T-cells are suppressed through accumulation of tryptophan-derived immunosuppressive metabolites (Mellor and Munn, 2008). As tryptophan is essentially provided through the diet, gut microbiota may help cancer cells to evade immunity in the human body through assimilation of available tryptophan in the gut.

## Kynurenine Pathway

**FIGURE 2:**
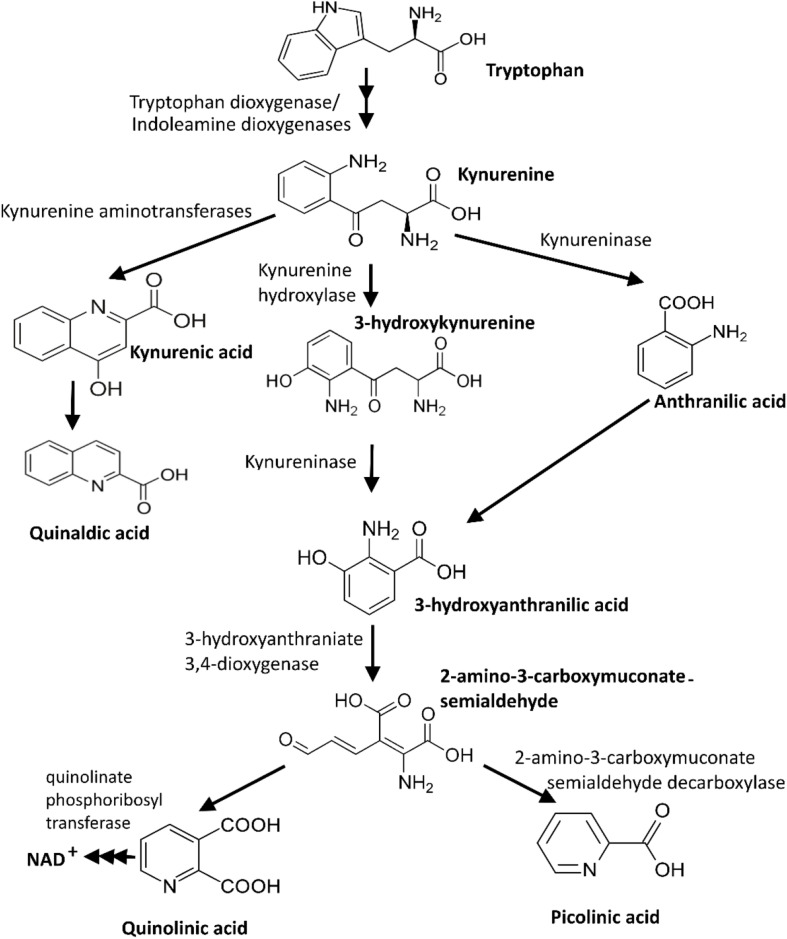
Kynurenine pathway, the main route of tryptophan catabolism (Dehhaghi et al., 2019a).

Tryptophan, an essential amino acid for animals and humans, is mainly supplied through dietary nutrient intake. The kynurenine pathway is the main route of tryptophan catabolism, contributing to *de novo* synthesis of nicotinamide adenosine dinucleotide (NAD) through the production and conversion of various neuroactive intermediates (Figure 2; Chen and Guillemin, 2009; Dehhaghi et al., 2019a). The heme-enzymes IDO-1, indoleamine 2,3-dioxygenase-2 (IDO-2), and tryptophan 2,3-dioxygenase (TDO-2) are three regulatory enzymes of the kynurenine pathway that catalyze the first step of tryptophan degradation. TDO and IDO are predominantly expressed in liver and various cells (e.g., intestinal cells, microglia, astrocytes, macrophages, and neuronal cells), respectively (Ball et al., 2014). IDO-1 expression is significantly induced by interferon gamma (INF-γ). However, other inflammatory cytokines, amyloid peptide, lipopolysaccharides, and TLR ligands could also induce its expression (Guillemin et al., 2003; Adams et al., 2012). The kynurenine pathway-derived metabolites, particularly kynurenic acid and quinolinic acid, could potentially have neuroprotective and neurotoxicity effects on the neuronal activity in the central and peripheral nervous systems, respectively (Carpanese et al., 2014; Filpa et al., 2015). Therefore, an aberrant kynurenine pathway metabolism could be triggered by unbalanced gut microbiota.

Clinical studies have suggested IDO-1 activity could be associated with various cancers including melanoma, colorectal, breast, lung, and brain cancers (Ferdinande et al., 2012; Zhai et al., 2015, 2018). Glioma and glioneuronal tumors show increased tryptophan uptake and catabolism. Compared to control healthy cells, the activity of IDO-1 is induced in malignant glioma cells due to the presence of INF-γ (Adams et al., 2012). This could induce pro-cancer activities through depletion of tryptophan (see Section “Tryptophan”). The upregulation of IDO-1 mRNA levels is positively correlated with the glioma grade, while it has an inverse relationship with the survival rate in patients with gliomas (Zhai et al., 2017). In fact, the overexpression of IDO-1 in cancer cells helps to evade immune surveillance by suppressing the expansion of T-cells and other immune cells.

Gut microbiota can influence the kynurenine pathway through the regulation of immune system-associated IDO-1 activity or the modulation of tryptophan availability. For instance, the levels of tryptophan circulation increase in the absence of gut microbiota (in germ-free models) or altered microbial composition induced by antibiotics (Dehhaghi et al., 2019a). This aberrant tryptophan level further decreases kynurenine-to-tryptophan ratio in plasma by modifying IDO-1 activity. Unlike the circulatory tryptophan level, both kynurenine metabolites and the peripheral serotonin level decrease in germ free animals (Clarke et al., 2013; Yano et al., 2015). It is important to note that the introduction of some gut microorganisms, for example *Bifidobacterium infantis*, can restore the normal kynurenine-to-tryptophan ratio (Desbonnet et al., 2008). This is because gut microbiota is capable of degrading tryptophan to various metabolites that consequently limit the availability of tryptophan for the kynurenine pathway and other tryptophan catabolism routes such as the serotonin production pathway.

Interestingly, IDO-1 and gut microbiota have feedback control on each other. While IDO-1 can induce an immunosuppressive response in the GI tract through the regulation of microbial metabolism and immune reactivity, gut microbiota can alter the kynurenine pathway and IDO-1 activity by affecting tryptophan availability (Dehhaghi et al., 2019a). Moreover, molecular analyses of bacterial genomes have revealed homologs of TDO, 3-hydroxyanthranilate-3,4-dioxygenase, kynurenine-3 monooxygenase, and kynureninase. These genes confer gut microbiota the potential to produce neuroactive metabolites. The microbial products of these homologs can influence the expression and activity of IDO-1.

Some microbial derived metabolites in GI such as SCFAs exert an anti-inflammatory effect and modulate the immune system and kynurenine pathway through regulation of IDO-1 activity. Generally, the interaction between cells and SCFAs activates a signaling cascade that involve G-protein coupled receptors (GPR41, GPR43, and GPR109a) (Brown et al., 2003; Dehhaghi et al., 2018a). SCFAs (especially, butyrate) that are produced by gut microbiota play a significant role in intestinal homeostasis and cancer protection. It has been demonstrated that butyrate-associated IDO-1 regulation is mainly conducted by two mechanisms that are independent of the known G-protein coupled receptors (i.e., GPR41, GPR43, and GPR109a) for SCFAs (Martin-Gallausiaux et al., 2018). The first mechanism involves the downregulation of the expression of signal transducer and activator of transcription (STAT) 1, which is a main mediator of IDO-1 expression. Decreased STAT1 expression inhibits INF-γ-dependent STAT1 phosphorylation, and subsequently reduces STAT1-dependant transcriptional activity of IDO-1. In the second mechanism, IDO-1 activity is affected through a STAT1-independent route for the inhibition of IDO-1 activity. In this mechanism, IDO-1 activity is regulated by SCFAs through their histone deacetylase (HDAC) inhibitory properties (Dehhaghi et al., 2018a). The inhibition of HDAC could suppress the production of proinflammatory cytokines such as tumor necrosis factor-α (TNF-α), IFN-γ, and IL-6 and could subsequently inhibit IDO-1 activity (Glauben et al., 2006).

## Gut Dysbiosis and Microglial Dysfunction

Immunity responses in the brain are controlled by microglial cells, i.e., resting microglia and activated microglia (viz., proinflammatory microglia, M1 phenotype; and immunosuppressive and tissue-regenerating phenotype microglia, M2 phenotype). The polarization process could be controlled by stimulus, period, and environment, which modulate the expression of CD86, CD45, MHC class II in microglia (Ma et al., 2017). More specifically, the resting microglia could be activated and polarized toward M1 phenotype through stimulation by trauma-induced cellular debris, bacterial-derived compounds (e.g., lipopolysaccharide), cytokines produced by type 1 T helper (Th1) cells and astrocytes (e.g., interferon-γ, and TNF-α). Many of these compounds are also produced or induced by gut microbiota (Heijtz et al., 2011; Dehhaghi et al., 2018a). M1 phenotype produces (i) proteolytic enzyme matrix metalloproteinase-3 and matrix metalloproteinase-9, (ii) redox signaling molecules such as reactive oxygen species and nitric oxide, and (iii) several proinflammatory cytokines, including IL-1ß, IL-6, targeting chemokine (C-C motif) ligand 2, TNF-α, C-X-C motif chemokine 10. Normally over time, type 2 T helper (Th2) cells stimulate the polarization of microglia into M2 phenotype through the production of IL-4 and IL-13. Unlike M1, M2 microglia produce scavenger receptors and anti-inflammatory cytokines, including tumor necrosis factor-β (TGF-β), insulin-like growth factor 1, IL-4, IL-10, and IL-13. Three phenotypes of M2 activated microglia function in inflammation suppression (i.e., M2a sub-class) and tissue regeneration (M2c sub-class), while M2b still has an unknown role (Latta et al., 2015; Mecha et al., 2015).

Therefore, Th 1 and Th 2 cells could mediate microglial phagocytosis, and their production of cytokines and other effector molecules that ultimately suppress the cause of inflammation. The disruption of these control mechanisms; however, could cause autoimmune diseases as well as brain cancer. The latter complication may be attributed to the unregulated tissue reconstruction triggered by activated M2 microglia. Switching to anti-inflammatory M2 phenotype, specifically M2c subtype, is induced by cytokines and corticosteroids produced by tumor cells, such as IL-10, TGF-β, and glucocorticoids. Accordingly, M2c phenotype is a deactivated form of microglia which is associated with tumor growth and brain malignancies (Mantovani et al., 2004; Roesch et al., 2018). More specifically, human high-grade gliomas have overexpressed amounts of cytokine colony stimulating factor 1 (CSF1), CSF1 receptor, and cytokines (i.e., IL-6 and TGF-β). It is worth mentioning that CSF1 is produced by microglia and macrophages, whereas IL-6 and TGF-β are released by Th 2 cells. TGF-β strongly inhibits cytokine production, lymphocyte proliferation, and T cell differentiation. In contrast, IL-2 and IL-12 are released from Th 1 cells in normal tissues and are absent in glioblastoma tumors (Mehrian-Shai et al., 2019; Figure 1).

Further, intestine epithelial cells produce high levels of TGF-β that is controlled by gut microbiota. For example, microbial fermentation products such as SCFAs (i.e., butyrate, acetate, and propionate) could be produced by *Clostridium* spp. (Dehhaghi et al., 2019a) even in gut, promoting TGF-β production (Bauché and Marie, 2017; Dehhaghi et al., 2019a). While the role of TGF-β in neuronal and glial cell development has been well documented, gut microbiota may play a crucial role in microglial maturation and function through controlling TGF-β production. This hypothesis has been supported by an *in vivo* study involving germ-free mice (Erny et al., 2015). Compared to the control normal mice, germ-free mice showed substantial changes in the properties of the microglia, including alteration in morphological features, gene expression, and the maturation process. Additionally, microbiota diversity is also correlated with the maturation of microglia. In fact, limited gut microbiota diversity could result in defective microglia (i.e., the number of immature microglia in the brain cortex increased) (Erny et al., 2015). It is worth mentioning that gut microbiota may also increase the severity of cancer by the production of neurotoxic substances (e.g., amyloid proteins, and lipopolysaccharides) that could pass BBB. Once in the brain, these microbial compounds increase ROS production by the activation and inflammation of microglia (Dehhaghi et al., 2018a). The induced inflammation then may trigger IDO-1 overactivation (see Section “Kynurenine Pathway”), tryptophan depletion (see Section “Tryptophan”), or MDSCs activation (see Section “Myeloid-Derived Suppressor Cells”).

## Myeloid-Derived Suppressor Cells

MDSCs are a heterogenous population of immunosuppressive cells that originate from immature myeloid cells. Following immature myeloid cells generation in bone marrow, they are released for their subsequent differentiation into mature myeloid cell, i.e., dendritic cells, macrophages, or granulocytes in peripheral organs. Unlike in a steady state, a partial inhibition in the differentiation of immature myeloid cells into mature myeloid cells occurs during various pathological conditions including cancer, and to a significantly lesser extent, infection, inflammation, sepsis, and trauma (Gabrilovich and Nagaraj, 2009; Raychaudhuri et al., 2015; Alban et al., 2019). These diseases expand MDSC in the circulatory system of patients by modifying STAT3 and Janus protein family members (Bromberg, 2002). Moreover, during pathological conditions, the activated immature myeloid cells remarkably express reactive oxygen species (ROS), nitrogen species (i.e., inducible nitric oxide synthase (iNOS) and nitric oxide), arginase 1, and other immune suppressive factors that increase the suppressive activity of MDSCs on T-cells in lymphoid organs, circulation, and tumors (in case of cancer) (Gabrilovich and Nagaraj, 2009).

In humans, CD11b^+^CD14^–^CD33, HLA-DR^–^-CD33, or HLA-DR^–^-CD15^+^ phenotypes of MDSCs have been identified (Gabrilovich and Nagaraj, 2009). MDSCs have the morphology of monocytes or granulocytes and can significantly suppress T-cell responses. Up to 5.4% of the total cells in GBM tumors is made up of MDSCs that mainly include lineage negative (CD14^–^CD15^–^), followed by granulocytic (CD14^–^CD15^+^) and monocytic (CD14^+^CD15^–^) subtypes (Raychaudhuri et al., 2015). The accumulation of MDSCs in GBM tumors attracts T regulatory cells but suppresses anti-tumor T-cell proliferation and functions. A positive correlation exists between the intratumoral density of glioma-associated MDSCs, and the patient’s survival and histological grade of gliomas (Gieryng and Kaminska, 2016). Intriguingly, T cell effector responses that are required for tumor rejection could be restored when MDSCs are removed (Movahedi et al., 2008; Gabrilovich and Nagaraj, 2009).

One of the promising methods for reducing the population of MDSCs is through enriching gut microbiota that increases granulocyte–macrophage colony-stimulating factor signaling (GM-CSF) (Brown et al., 2017). Moreover, gut microbiota can block STAT6 in MDSC by decreasing the expression of IL-4 that is important for signaling downstream of IL-4 receptor α-chain. In fact, this deficient signaling pathway prevents both the activity and production of arginase 1, which in turn, decrease the T-cell suppressive function of MDSCs (Rutschman et al., 2001; Gabrilovich and Nagaraj, 2009). It is worth mentioning that the IL-4 receptor α-chain-STAT6 pathway does not induce tumor immunosuppression in all tumor microenvironment.

Gut-microbiota can also decrease the suppressive activity of MDSCs through modification of ROS level. The generation of the ROS may occur during physiological processes (i.e., endogenous ROS), interaction with external harmful stress (exogenous ROS) such as ionizing radiation, or in a response to disease state (Dehhaghi et al., 2019c). ROS examples include nitric oxide and peroxynitrite, singlet oxygen, hydroxyl radicals, superoxide, and peroxides (Dehhaghi et al., 2019c). The speculative intervention of gut microbiota in the ROS level of the brain could be through the production of microbial metabolites (Bonaz et al., 2018; Dehhaghi et al., 2019a). Some of these metabolites such as SCFAs (see Section “Kynurenine Pathway”), vitamins, antioxidants, and polyphenols inhibit the ROS. The existence of gut microbiota could suppress extensive colonization of pathogens in the gastrointestinal tract while could also regulate the immune response and the permeability of BBB and intestinal barrier (Dehhaghi et al., 2018a). Some of these modifications and interferences may lower the generation of ROS in the body, even in the brain, and hence, inhibit MSDCs suppressive activity. Gut microbiota could also manipulate central nervous system ROS flux through providing extra amounts of some neurotransmitters (e.g., gamma-amino butyric acid, serotonin, and dopamine) and/or through kynurenine pathways (see Section “Tryptophan”).

On the downside, gut microbiota may also increase the severity of cancer through activation of microglia and the subsequent induction of ROS generation (see Section “Myeloid-Derived Suppressor Cells”). Gut microbiota could also increase the flux of nitric oxide (see Section “Arginine”) and peroxynitrite in the body and the brain. Excessive amounts of peroxynitrite render T-cells unresponsive, which could be associated with the tumor progressions in many cancer types (Bentz et al., 2000; Cobbs et al., 2003; Gabrilovich and Nagaraj, 2009). This could be attributed to the ability of peroxynitrite for modifying the antigen-specific stimulation response of T-cells due to aberrant nitration of the T-cell receptor and CD8^+^ molecules (Nagaraj et al., 2007; Gabrilovich and Nagaraj, 2009).

Alternatively, gut microbiota can induce the expression and activation of INF-γ following the assimilation of gluten which cannot be completely digested by humans (Dehhaghi et al., 2019a). INF-γ may inhibits anti-tumor T-cells through the expression of iNOS, arginase by MDSCs in the tumor microenvironment. However, further study has shown that although INF-γ has the potential to signal through the STAT1 pathway, it is not a key determinant for impacting T-cell suppressive potency, accumulation, or phenotype of MDSCs (Sinha et al., 2012). Therefore, gut microbiota-induced IFN-γ probably increases cancer pathogenesis through modification of the kynurenine pathway at IDO1 step (see Section “Kynurenine Pathway”).

## Conclusion

Gut-brain axis may negatively or positively influence cancer development, including brain cancer, through the production of various metabolites. These metabolites may induce or suppress the release of specific cytokines, which could trigger inflammatory or anti-inflammatory responses. The intervention of gut microbiota in cancer development can be both negative and positive. For example, gut microbiota can modulate suppressive activity of MDSCs on anti-tumor T-cells by producing pro-inflammatory (e.g., amyloid proteins and lipopolysaccharides) and anti-inflammatory substances (e.g., antioxidants, polyphenols, and vitamins). With respect to anti-tumor activities, some gut microorganisms such as *Lactobacillus reuteri, Enterococcus faecalis, Lactobacillus crispatus* and *Clostridium orbiscindens* can induce the differentiation of MDSCs into mature myeloid cells. This is done by mediating the release of GM-CSF that is controlled by IL-17A. Individuals with a high population of *Lactobacillus* spp. in their GI track show a lower expression of IL-4 that suppressed the activity of MDSCs on anti-tumor T-cells by downregulating arginase 1 expression.

Through depletion of some dietary amino acids, gut microbiota can modulate the tumor microenvironment. On this basis, the assimilation of dietary arginine in the gut by microorganisms (e.g., *Prevotella* spp.) reduces flux in the human body that favors anti-tumor activities. Under an arginine depleted condition, arginine-auxotrophic cancers cells must extensively rely on autophagy to meet their rapid metabolisms that eventually trigger apoptosis. As a result of reduced arginine flux, a lower level of nitric oxide is synthesized by the body that inhibits the expansion of MSDCs, and hence, improves the activity of the anti-tumor T-cell. However, microbial derived nitric oxide could be also produced during the assimilation of dietary arginine by gut microbiota. The resultant nitric oxide could then form peroxynitrite upon its reaction with superoxide radicals. Both nitric oxide and peroxynitrite can induce tumor development by increasing MSDC-mediated suppressive activity on T-cells, whereas polyamines (another group of arginine-derived microbial compounds in the gut) induce the proliferation and metastasis of tumor cells. Alternatively, peroxynitrite causes pro-cancer activity through rendering T-cells unresponsive due to aberrant nitration of the T-cell receptor and CD8+ molecules. Nevertheless, it should also be noted that tumor cells are more prone to nitric-oxide-mediated cellular damages (i.e., mitochondria and DNA) and apoptosis due to their aberrant P53 pathway.

Unlike arginine, the depletion of tryptophan by gut microbiota (e.g., *Burkholderia, Ralstonia, Klebsiella, Citrobacter*, and *Bifidobacterium infantis*) induced tumor immune-resistance and survivability by the phosphorylation of eukaryotic initiation factor 2α. Moreover, fatty acid synthesis in human primary CD4+ T-cells is disrupted due to the overactivation of GGN2 in tryptophan depleted environments. This leads to aberrant proliferation and functions of effector T-cells in favor of tumor cells. Gut microbiota may also convert tryptophan into kynurenine and kynurenic acid that over-activate AhR. Ligand-activated AhR then can induce IDO-1 activity through promoting the release of IL-6 and INF-γ. IDO-1 could also be overactivated due to the production of inflammatory cytokines, amyloid peptide, and lipopolysaccharides. Interestingly, IDO-1 overactivation leads to further tryptophan depletion. In contrast, gut microbiota may also produce anti-inflammatory products such as SCFAs that inhibit both INF-γ and IDO-1 by downregulating STAT1 and histone deacetylase. However, SCFAs could also induce the release of TGF-β that triggers dysbiosis of Th1 and Th2 in favor of microglia M2c phenotype and inhibits cytokine production, lymphocyte proliferation, and T cell differentiation.

## Author Contributions

MD and HK wrote the manuscript and prepared the figures. GG and BH have reviewed and corrected the manuscript based on their respective expertise. All authors read and approved the final version of the manuscript.

## Conflict of Interest

The authors declare that the research was conducted in the absence of any commercial or financial relationships that could be construed as a potential conflict of interest.
